# Adolescents’ experiences of being a relative of a critically ill patient in an intensive care unit: a qualitative study

**DOI:** 10.1186/s12913-026-14632-w

**Published:** 2026-04-30

**Authors:** Mathilde Elsa Christensen, Gudrun Elin Rohde, Gro Frivold

**Affiliations:** 1 Department of Health and Nursing Sciences, University of Agder, Jon Lilletuns vei 2, Grimstad, 4879 Norway; 2 Department of Health and Nursing Sciences, University of Agder, Gimlemoen 25, Kristiansand, 4630 Norway

**Keywords:** Adolescent, Critical illness, Family relations, Intensive care units, Nursing, Qualitative research, Relatives

## Abstract

**Background:**

Existing literature suggests that adolescents often find critical illness overwhelming and receive inadequate support and information from both parents and healthcare professionals.

**Objective:**

To explore the experiences of adolescents as relatives in an intensive care unit (ICU).

**Design:**

This study utilised a qualitative approach with an exploratory and descriptive design with in-depth interviews.

**Participants:**

Sixteen adolescent relatives of critically ill patients were recruited from three different ICUs in Norway.

**Methods:**

The study utilised semi-structured in-depth qualitative interviews to gather data, which were subsequently analysed using reflexive thematic analysis.

**Results:**

We identified two main themes, and five subthemes. 1. Life turns upside down: exploring a threat to life’s security; (a) Alone in uncertainty during a relative’s critical illness, and (b) Seeking clarity and guidance to navigate the impressions from a transformed reality. 2. The significance of an inclusive environment; (a) Feeling invisible and excluded, (b) The need to be near and being prepared, and (c) Feeling involved by being informed.

**Conclusions:**

Adolescent relatives of critically ill patients experience significant uncertainty and distress. The ICU environment is perceived as intimidating and technical. They frequently feel overlooked and in need of a welcoming and supportive environment. By individually preparing adolescents before their initial visit, healthcare professionals can better meet their responsibilities, minimise trauma, and contribute to a sense of belonging for adolescent relatives in the ICU. Care can be improved by addressing their needs and providing tailored information. Health authorities should prioritise resource allocation to support family-centred care practices and ensure that these objectives are achieved.

**Clinical implications:**

Understanding adolescents’ experiences when a relative is admitted to an ICU is essential for all healthcare professionals who interact with critically ill patients and their families. This study suggests that adolescents need to feel a sense of belonging within the ICU and underscores the importance of systematic preparation and tailored communication in ICUs to adequately support and involve adolescents.

**Supplementary Information:**

The online version contains supplementary material available at 10.1186/s12913-026-14632-w.

## Background

Adolescents who are relatives of critically ill patients admitted to an intensive care unit (ICU) often receive less focus and support than adult relatives [1, 2]. This presents a paradox, as adolescents are similarly thrust into unexpected existential crises and experience profound shock due to the traumatic circumstances of critical illness in the family [1, 3]. An ICU treats critically ill patients at a high medical standard, involving numerous technical installations and a hectic environment where many healthcare professionals (HCPs) work closely together, often lacking separate rooms for each patient [4]. Such an environment can be perceived as intimidating and unfamiliar to those who come from outside [4, 5].

Trauma is defined as ‘an emotional response to a terrible event like an accident, crime, natural disaster, physical or emotional abuse, neglect, experiencing or witnessing violence, death of a relative, war, and more’ [6]. Traumatic experiences during adolescence can increase the likelihood of a range of adverse outcomes [7]. Therefore, addressing traumatic experiences during this period holds substantial significance.

Previous research shows that adult relatives are exposed to significant emotional stress when a critical ill patient is admitted to the ICU, which can have a negative impact on their lives and health [8, 9]. The most critical needs they have include the need for comprehensive information, proximity to the patient, care and support from the ICU staff [10, 11]. Adult relatives often find the high-tech ICU environment to be stressful and frightening [12]. After the ICU admission, adult relatives often feel powerless and experience a wide range of psychological burdens, such as depression, anxiety, impairments in quality of life, and posttraumatic stress disorder (PTSD; [13]. These symptoms are known as post-intensive care syndrome-family (PICS-F; [14].

As part of holistic care in the ICU, family-centred care (FCC) is an approach that recognises the essential role relatives play in the health and recovery of patients [15], acknowledging the specific needs of families, and the importance of preventing negative long term health consequences [16]. FCC emphasises collaboration and communication between HCPs and relatives, considering their needs and preferences while ensuring patient-centred care [17]. Moreover, FCC involves respect, participation, information-sharing, and collaboration [18] and is a quality standard in intensive care, engaging relatives as decision-making partners [16–18]. However, FCC has been criticised for excluding children and adolescents, focusing primarily on HCP–parent/adult relations [19].

Existing research on adolescent relatives in ICUs is sparse and frequently combines children and adolescents into a single group. Consequently, previous research is presented here as pertaining to both children and adolescents. For children and adolescents, the impact of a relative being admitted to the ICU is especially profound, due to their experiences of physical and psychological distance [1, 3]. A meta-synthesis of their experiences during a parent’s critical illness and ICU admission reveals that the situation is overwhelming [1]. To comprehend and manage the experience, children and adolescents seek information that aligns with their individual capacities. They express a strong need to remain close to their ill parents and to be recognised and treated as integral members of the family. When they are not involved, children and adolescents often feel overlooked and unsupported before, during, and after the ICU stay [1].

Visiting the ICU can help children and adolescents gain a better understanding of their relative’s condition and maintain family connections, despite the potentially traumatic experience. HCPs can help mitigate trauma by explaining ICU functions, preparing children and adolescents for the environment, facilitating caregiving opportunities, and ensuring privacy [20]. In addition, recognising the cognitive and relational benefits of visitation may motivate HCPs to develop creative ways to maintain connections during critical care and separation [3]. Many countries still have strict visiting policies, even though visits can help children and adolescents understand the situation, foster closer relationships, and alleviate feelings of uncertainty [21]. Symptoms of depression and anxiety are observed in children and adolescents regardless of visitation, indicating that individual characteristics and ICU dynamics significantly affect outcomes [22]. Those who do not visit report more regret and increased PTSD symptoms. Visits should be flexible, thoroughly prepared, and tailored to the children and adolescent’s previous experiences and stage of development [20]. Children and adolescents feel positive when visiting their ill parent in the ICU, yet they often do not feel fully involved and desire a more compassionate, caring approach from critical care nurses (CCNs). Improvements are needed in how CCNs engage with visiting children in a more individualised and caring manner [23]. There is a lack of consistency in the application of policies and procedures to facilitate children visiting their relatives in the ICU [24].

Children’s rights as dependents in hospitals were strengthened by the 2010 amendment to the Health Personnel Act and the Specialist Health Services Act [25]. These amendments are embedded in § 10a of the Health Personnel Act, which outlines the duty of HCPs to support minor children who are dependents of parents or siblings, and § 3–7a of the Specialist Health Services Act, which mandates healthcare institutions within specialist health services to employ personnel responsible for children’s welfare as needed.

An exploratory cross‑sectional multicentre study conducted in Norway revealed that while routines mandated by legislation are improving, the law is still not functioning as intended [26]. In addition, Frivold, Lind [27] investigated the experiences of CCNs with child welfare roles and found that there is room for improvement in this area. Systematic follow-up is crucial and should be integrated into future holistic care strategies [28].

Existing research have focused primarily on the experiences of adult relatives, leaving a significant gap in our understanding of how adolescents are affected during a relative’s admission to the ICU [21]. Acquiring new insights into these adolescent experiences is crucial for developing appropriate care interventions to address their unique needs. To our knowledge, no studies have yet explored the experiences of Norwegian adolescents as relatives of ICU patients.

This study therefore explores adolescents’ experiences of being relatives in the ICU context.

## Methods/methodology

### Design

This study utilised a qualitative approach with an exploratory, descriptive design, incorporating semi-structured individual qualitative interviews with adolescent relatives of critically ill patients [29].

### Study setting and recruitment

This study is part of a larger research project focused on adolescent relatives of critically ill patients admitted to an ICU. Adolescents were recruited by hospital contacts from three different hospitals with five general ICUs in Norway. The hospitals were at regional and university level covering three regions with a combined population of 1.3 million people. In the electronical survey study, there was an invitation to participate in an interview study at the end of the questionnaire. Most adolescents were identified through the contact information of former ICU patients, where underaged children as relatives were registered and they were recruited by mail [25, 30]. Hospital contacts were dedicated CCNs who worked in the respective ICUs and obtained the necessary research ethics and legal approvals to recruit participants for the interview study. Letters containing information about the project and details for participation were sent to the adolescents’ postal addresses. To adolescents under 16 years of age, a dedicated letter to their parents was included. Participation was voluntary, and interested adolescents were asked to contact the project leader or the project’s doctoral student directly.

To ensure a purposeful sample of participants, hospital contacts assessed the impact of the pandemic on adolescent relatives. As a result, half of the invitation letters were sent to adolescents who had experienced the ICU before the COVID-19 pandemic, while the other half were sent to those who had such experiences during the pandemic. These hospital contacts, serving as local project liaisons, were tasked with distributing information on the study. We encountered challenges with recruitment and expanded our efforts to include recruitment via social media. Despite similarities in gender and age, we chose to include all 16 adolescents who expressed a desire to participate in the study. Thus, the recruitment can be considered more as convenience-based rather than purposive. Recruitment requests were sent out from April to September 2022. The adolescents were provided with both written and verbal information prior to the interviews being conducted.

### Inclusion and/or exclusion criteria. definition of concepts

This study initially included adolescents aged 11–18 years at the time of admission. To be eligible, participants had to have experienced the discharge of a critically ill patient between 4 weeks and 2 years prior. Two participants were included despite falling outside the inclusion criteria concerning age and the time elapsed since admission. The term ‘adolescent’ was applied in line with the World Health Organization’s (WHO) definition as the phase of life between childhood and adulthood, spanning from age 10 to 19 [7].

### Data collection

Between May 2022 and February 2023, the first author conducted a total of 16 in-depth interviews with the presence of only the researcher and the adolescent. The interviews, lasting from 31 to 76 min (with a median of 45 min), were audio-recorded. They were conducted at various locations chosen by each adolescent, including their homes and schools, with one conducted digitally face-to-face. The adolescents chose if they would have their parent(s) with them during the interview. None of them chose to have their parents present. All interviews were transcribed verbatim by the first author, establishing a strong connection between the researcher and the data [29].

During the interviews, the first author was particularly attentive during the interview situation, as these were young vulnerable individuals, and there was a significant imbalance in the power dynamics between the researcher and the interviewee. Since talking about traumatic events could be distressing, a support plan was created to offer assistance if the adolescents needed further help after the interview. This plan involved providing access to easily accessible services aimed at helping adolescents cope.

The interview guide (Table 1), included semi-structured open-ended questions, such as, ‘Can you tell me about your first encounter with the ICU?’ Reflexive dialogue was encouraged with open questions such as, ‘Tell me more,’ and ‘Could you explain further?’ [29].

**Table 1:** Interview guide

The interview will begin with an open question encouraging participants to freely share their experiences regarding the stay and their thoughts on what the best care in the ICU would be.
What had happened to the person who was ill? The first encounter with the department.
How did you experience being cared for by the nurses and doctors? (support, care, and presence).
How were you informed about what had happened and what was going to happen? (information).
How did you perceive the facilities and the opportunity to be present? (facilitation).

### Data analysis

Interview transcripts were analysed using Braun and Clarke’s reflexive thematic analysis (RTA; [31]. Reflexive thematic analysis was chosen because it allowed the researcher to be reflexive and engage with the dataset within the paradigm of interpretation. Throughout the analytic process, RTA enabled the first author to be responsive and flexible, allowing the data to shape the results through the six phases of the analytic process, resulting in a robust analysis. The six phases of RTA included: familiarisation with the data, generating initial codes, searching for themes, reviewing themes, defining and naming themes, and producing the report [31].

The first author immediately noted her thoughts after the interviews. The first author read the transcripts and listened to the audio several times to fully understand the text and capture any important insights. They recorded relevant observations and ideas about the content. The co-authors read the transcripts, and the interviews were then imported into NVivo 14 [32].

The first author, supported by the co-authors systematically analysed the dataset, assigning code labels to highlight significant data segments. Code labels were discussed among the co-authors. All interviews were coded across cases before generating initial themes. We then returned to code further to develop comprehensive codes and themes. During the analysis, the user representative was engaged to review and discuss the coding process and the development of initial themes. This contribution was based on her previous experiences as a relative of a critically ill brother in the ICU. Throughout the writing process, all authors contributed and reached consensus on finalising the naming of the themes.

Codes were generated by the researchers in NVivo 14 software. All potential concepts that emerged were noted with a view to gaining an understanding of the data’s significance and developing themes to address the research question.

The COREQ standard for reporting qualitative research was followed to ensure a rigorous methodology and is added as a supplementary file [33].

### Ethics approval and consent to participate

The invitation for adolescent participation included up to three information letters: one for adolescents aged 11–15 and one for their parents, as parental consent was required up to the age of 16. A separate letter, written in more mature language, was provided for adolescents aged 16–18, along with their own consent form. An additional letter was available for the affected third party, such as the former ICU patient. These letters were drafted in accordance with guidelines from the Norwegian Centre for Research Data to ensure compliance in recruiting adolescents for scientific research. All adolescents, along with the parents of those under 16 years of age, provided written informed consent for participation in the study.

The study adhered to the ethical research principles outlined in the Helsinki Declaration [34]. Ethical approval was provided by the Regional Committees for Medical and Health Research Ethics (no. ******), the Norwegian Centre for Research Data (no. ******), and the Norwegian National Committees for Research Ethics in the Social Sciences and the Humanities. In addition, ethical approval was granted by the collaborating hospitals involved in the recruitment.

### Rigour and reflexivity

Maintaining reflexivity throughout the research process demonstrated how the researcher’s role influenced the analysis and how interpretations were rooted in a deliberate and critical approach [31]. The reflexive notes strengthened the credibility and transparency of the analysis, as did the quotes that validated the interpretations. We provide comprehensive and transparent details regarding participant characteristics, research context, and the analysis process. During the entire project all authors have worked closely which further increase the reflexivity and rigour in the study.

The first author is a female Ph. D candidate and seasoned CCN with wide-ranging experience in adult ICUs and neonatal ICUs, including the child welfare role. Both co-authors are distinguished for their extensive and robust clinical experience in specialist health services, holding Ph. D degrees, and serving as professors in Health Sciences. One specialises in health-related quality of life, while the other focuses on research concerning family caregivers in the ICU context, emphasising their contributions to their respective fields.

The epistemological stance is hermeneutic phenomenological, with the aim of comprehending the depth of adolescents’ personal experiences as relatives in ICU.

### User representations

The user representative is a 26-year-old former relative of a critically ill ICU patient during her adolescence. Throughout all stages of the project, she was involved and contributed to relevance to the target group. She attended meetings and provided important input on how this phenomenon was experienced from the user perspective.

By including a user representative, we pursued that the perspectives and needs of the target group were effectively integrated, thereby enhancing the outcomes’ relevance, accessibility, and overall impact [35].

The recruitment of the user representative was facilitated by one of the supervisors, who identified her interest in the subject matter. Subsequently, the supervisor proposes her for the role of user representative.

## Results

Sixteen adolescent relatives of ICU patients were recruited for the study. The adolescents were relatives of critically ill parents, grandparents and siblings. Characteristics of the adolescents is presented in Table 2.

**Table 2:** Characteristics of adolescents in the study

Number	Name	Current Age (yrs)	Gender	ICU Age (yrs)	Patient survival	Relation to the patient	ICU stay
1	Mari	18	Girl	16	Yes	Daughter	3 days
2	Hedvig	16	Girl	14	Yes	Daughter	3 days
3	Sissel	14	Girl	13	Yes	Daughter	7 days
4	Kaja	12	Girl	11	Yes	Daughter	7 days
5	Elisabeth	17	Girl	17	Yes	Sister	5 days
6	Jenny	18	Girl	14-15-16	Yes	Daughter	Several stays
7	Nora	16	Girl	16	Yes	Grandchild	14 days
8	Filippa	19	Girl	0–19	Yes	Daughter	Several stays
9	Tobias	17	Boy	0–17	Yes	Son	Several stays
10	Elvira	17	Girl	8	Yes	Daughter	14 days
11	Irene	14	Girl	13	No	Grandchild	14 days
12	Einar	12	Boy	11	No	Grandchild	14 days
13	Bente	17	Girl	16	No	Grandchild	7 days
14	Marthe	15	Girl	7-10-13	No	Daughter	Several stays
15	Torvald	17	Boy	16	Yes	Son	7 days
16	Klara	14	Girl	13	Yes	Daughter	7 days

Pseudonyms were assigned to the participants by the first author. Each participant’s pseudonym is given in the quotes, along with their current age in parentheses.

In the course of reflexive thematic analysis, we identified two themes with five sub-themes (Table 3).

**Table 3:** Reflexive thematic analysis process

THEMES	SUB-THEMES	CODES examples	QUOTES examples
Life turns upside down: Exploring a threat to life’s security	Alone in uncertainty during a relative`s critical illness	Being left alone Status update not given	*‘Suddenly*,* I was completely alone at home. I just remember mum saying that auntie would be coming soon*,* so just stay here and everything. And then I wasn’t told anything more.’ Mari*,* (18).*
	Seeking clarity and guidance to navigate the impressions from a transformed reality	ICU patient looks ICU environment affects	*‘And it was a bit*,* it was a bit unsettling to see mum and all those machines*,* and she just lay there. She looked just like she was dead.’ Kaja*,* (12).*
The significance of an inclusive environment	Feeling invisible and excluded	Feeling of not being seen Not welcomed	*‘We were hardly greeted when we arrived. It seemed they didn’t even say “Hello” as we came in. I think we simply walked in.’ Nora*,* (16).*
	The need to be near and being prepared	Prepared by health care professionals Prepared before visit	*‘And then they took us into a room and gave us information*,* explaining what was going to happen. And then we went in. When I was in that room*,* I was very impatient because I wanted to see mum. I didn’t like being there very much*,* because I was so impatient*,* but it was actually a good initiative. Erm. Then I went in.’ Sissel*,* (14).*
	Feeling involved by being informed	Information given by healthcare professionals Feeling safe	*‘But we talked to someone at least. Then they said that she would most likely be okay. So*,* I thought like ‘okay*,*’ and I wasn’t so afraid after that.’ Sissel*,* (14).*

### Life turns upside down: Exploring a threat to life’s security

The adolescents perceive having a relative in the ICU as a life-altering event, as it threatens the sudden loss of someone dear and disrupts their daily routines. Up until this point, they have mostly lived predictable lives, and this experience therefore provokes concern and uncertainty about both their present and future. During these periods, adolescents undergo a tumultuous emotional journey, marked by uncertainty, fear, and hope. They all experience a transformative shift, clearly marking a before and after their relative’s critical illness.

#### Alone in uncertainty during a relative`s critical illness

Many adolescents find themselves facing a close relative’s sudden and severe illness while at home or school. When a relative needs sudden hospitalisation, the adolescents are left to cope with the situation on their own, as their parents are either the ones who are critically ill or are busy at the hospital. These adolescents may be left at home alone, anxiously waiting for news, or placed in the care of neighbours, grandparents, or friends. This experience brings about feelings of isolation and confusion about the illness and its implications, especially when they do not fully grasp the seriousness of the situation.

1: ‘I just remember Mum saying that Aunt is coming soon, so just stay here and all. And then I didn’t hear anything after that.’ Mari, (18).

Many adolescents witness a relative being taken by ambulance from home, experiencing a sense of urgency and responsibility despite feeling scared and isolated. In the upcoming quotation, Jenny explains how she was left alone with her two sisters to clean up after the ambulance had left. Adolescents understand the need to be independent in such situations, but they struggle to cope without the support of their parents.

6: ‘I understood it was serious, and I saw several of the seizures. I especially remember one time; the ambulance came to our house because she had a seizure, and I think Dad wasn’t home. Once they took her to the hospital, they left a lot of needles and medical stuff behind, which we cleaned up because they just had to get her to the hospital. And so, we walked around and there were dirty footprints everywhere from the ambulance people because they had to hurry… it was really hard. Both for me and for my sisters.’ Jenny [18], .

Adolescents grapple with numerous unanswered questions, eagerly awaiting updates on their sick relative’s condition. However, hours can pass without any information, until they eventually receive an update, typically from their parent. This prolonged uncertainty exacerbates their emotional turmoil, underscoring the critical need for effective communication and support when adolescents are left alone while their parents are at the hospital. Another perspective on being alone is when adolescents are not informed that their relatives have been taken by ambulance or when they feel that the adults in the family downplay the severity of the situation. In these instances, they feel disregarded as integral family members, leading to increased frustration towards adults and parents and increased fear for their critically ill relative.

#### Seeking clarity and guidance to navigate the impressions from a transformed reality

Adolescents often struggle to comprehend the gravity of a situation, until they find themselves facing reality in the ICU. Entering the ICU intensifies their feelings of uncertainty, fear, and concern. The ICU setting is both intimidating and overwhelming. The high-tech environment makes the ICU seem alien and very scary. Adolescents perceive the ICU as a white, cold, sterile, technical, and eerie place, appearing closed off from them. This is due to the unit being locked to outsiders and feeling like ‘a different world’. The emphasis on the critically ill patient underscores the severity of the illness. The infection control attire worn by ICU staff in isolation rooms can be quite intimidating to adolescents. This attire includes full-covering yellow gowns, hoods, visors, face masks, and long gloves, which make the ICU staff appear unrecognisable. These impressions can be overwhelming for adolescents, causing them to perceive the ICU as vastly different from other hospital wards. The sombre atmosphere is further intensified by the presence of other critically ill patients in the ICU, leaving adolescents with a strong impression that the ICU is a place where people go to die.

Many of the adolescents characterise seeing their relatives as a profound, eye-opening experience that serves as a stark reality check for which they are mentally unprepared. Adolescents frequently perceive their relatives as alien and unfamiliar, describing them as looking lifeless or dead. Encountering a comatose patient, often ventilated and attached to numerous medical devices, elicits feelings of estrangement and fear. The most significant impression cited by adolescents is the drastic change in the patient’s appearance and condition, with many finding their physical expressions markedly different.

2: ‘And then we went in, and there were these big doors, so I was like, ‘Oh, okay.’ It was kind of weird – there were lots of people, and he was lying there. It was very weird because he had lots of cords connected, and I almost didn’t recognise him because he was full of bruises and lots of stuff. So it was just very, very strange. Then I got totally dizzy and really pale, and I felt like I had to throw up.’ Hedvig [16], .

Adolescents sometimes spend time in the waiting room of the ICU. They observe that the waiting room serves as a gathering place for the immediate family during the acute phase of ICU hospitalisation and as a place to receive information from HCPs. They often feel that the waiting room is not suited to their interests or age group. It is essential for them to receive updates from HCPs to ensure that their waiting time is not perceived as pointless.

1: ‘I just remember that in the little waiting room – I felt it was so small when we sat there waiting. I was with Mom and my half-sister. It was so boring to wait there because there was nothing there, in a way. I noticed that there weren’t any things there that I was interested in. There were either women’s magazines or home magazines, or there was Lego for little kids.’ Mari [18], .

### The significance of an inclusive environment

Adolescents need to feel that they are being taken seriously by adults and HCPs in the ICU. Trust is built when they are involved in conversations and decisions that are relevant to them. They seek a safe environment where they can express themselves without fear of judgement, as well as a fundamental sense that their everyday life remains stable despite the situation. The need for proximity to the patient and other relatives is particularly strong, as it serves to affirm bonds and provide emotional support. Maintaining these relationships is important for adolescents, helping them feel less isolated and more understood during their ICU experience.

#### Feeling invisible and excluded

HCPs and adult relatives tend to focus their attention primarily on the patient, which can lead adolescents to feel overlooked in communication dynamics. In interactions between HCPs and parents, adolescents may feel excluded as they feel parents withhold the truth about the situation, and HCPs mainly address the parents or other adult relatives. Adolescents believe that this dynamic may stem from adults’ biases regarding their ability to handle honest information. As a result, this worsens their feelings of isolation within the ICU setting. Moreover, adolescents experience a lack of access to information, as adults routinely exclude them from critical updates and shield them from significant details.

10: ‘So, there is a lot of confusion and sensitivity, and I don’t really know where I stand in the situation. When you go to family, they don’t know what to do. And when you go to the doctors, they won’t tell you anything. It’s kind of like you’re in the middle of everything, but outside of it all.’ Elvira [17], .

Many adolescents report not feeling welcome in the ICU. This sentiment arises when they are not greeted or even looked at upon arrival, adversely affecting their emotional well-being during an already challenging time. Such experiences contribute to feelings of invisibility and heighten their sense of not being valued or important.

1: ‘It often happened that they just talked to Mom to give messages and such. Nothing with me, in a way. So, it was kind of like she wasn’t really there for me then. Like she was a bit sidetracked. And I’m not that fond of talking to strangers, so I don’t take the first initiative, in a way.’ Mari [18], .

The availability of both parents within the family often changes as a consequence of having a relative in the ICU. Adolescents frequently find themselves in a situation where both parents are preoccupied with the hospitalisation, leaving them feeling uncertain and waiting for an update on the situation. In these moments, they feel at a loss, not wanting to demand too much of their parents, but also feeling excluded.

#### The need to be near and being prepared

Adolescents perceive their relationship as strong and feel strong empathy for their relative who have fallen ill. The bond significantly influences their reactions and the depth of their emotional response to their relative’s illness. Being close to the ICU patient, touching them, and knowing that they might hear what is being said is important for many adolescents.

4: ‘Yes, one of the nurses says that she might hear what we are saying. I don’t know if she did, but we gave it a try.’ Kaja [12], .

Many adolescents experience that the ICU patient displays different and more sensitive sides when they are in a crisis. This fosters closeness and makes the adolescents feel like an important caregiver.

Adolescents need to be supported and prepared for the ICU experience, both practically and emotionally. They require comfort and encouragement from family, friends, healthcare professionals, and other relevant figures. They express varying desires to visit their relatives in the ICU.

Adolescents often find themselves excluded from direct involvement due to ICU protocols limiting visitor numbers, which can lead to prolonged periods of inactivity and uncertainty. Some adolescents consciously choose not to visit their relative in the ICU to protect themselves emotionally, fearing increased distress. Those who decide against visiting for self-protection often express regret over this decision in hindsight. In this study, two of these adolescents were shown a photograph of their critically ill relative, which they found to be a helpful alternative.

Only a few adolescents are adequately prepared before entering the ICU. Preparation fosters a sense of trust between adolescents and the ICU staff, making them feel included and valued.

3: ‘They took us into a room and gave us some information on what was going to happen. And then we went in. When I was in that room, I was very impatient because I wanted to see mom. I didn’t like being there much, because I was so impatient. But it was a really good measure.’ Sissel [14], .

Furthermore, being prepared enhances their sense of belonging within the family and provides a measure of security during an already challenging time. While some adolescents feel prepared after a short briefing with HCPs, others need more detailed preparation.

#### Feeling involved by being informed

Being well-informed allows adolescents to feel secure and build trust with healthcare professionals, thereby facilitating improved communication and cooperation.

Adolescents prefer to receive information from HCPs such as doctors or CCNs; not from parents who are in a crisis and might lack the appropriate knowledge or communication skills.

3: ‘But then she got worse, and she was put under anaesthesia. No, she was put in an induced coma. At first, I was a bit scared, but then we went into a room where they talked to us. We spoke with Dad first and then with the doctors. I can’t remember exactly…But we talked to someone at least. Then they said that she would most likely be okay. So, I thought like ‘okay,’ and I wasn’t so afraid after that.’ Sissel [14], .

In this study, all adolescents concealed their true feelings and needs from their parents. They believed that their parents had enough to deal with, as well as limited capacity. They preferred having focused conversations, either alone or with siblings present, as it allowed them to ask questions freely without fear of causing their parents distress. They felt that information should be honest, customised to individual needs, and delivered by trained professionals in accessible language.

9: ‘Yes, if you’re simply included and given a very clear explanation: “Here is the problem, and this is a potential solution.” Whether it’s penicillin, surgery, or whatever. You need to be told what can happen going forward. I think it’s very important to focus on getting to know what the chances are for the future. Like, there is a very high probability it will go poorly, or it is almost guaranteed it will go well.’ Tobias [17], .

In this study, the adolescents proposed several strategies to enhance their sense of being informed. They suggested access to educational materials about bodily functions and images of typical ICU patients, to better understand their relative’s situation. They recommended that HCPs use dolls and visual aids to explain medical equipment and conditions and suggested placing a notice on the ICU door to indicate that critically ill patients are inside.

## Discussion

In this study, we examined adolescents’ experiences when a relative was admitted to the ICU. The adolescents experienced their relative’s critical illness as a traumatic event that disrupted their secure life. Seeing their relatives in the ICU served as a reality check for which they were unprepared, and many described it as a shock. All expressed a desire to be prepared and to receive individually tailored information. Across all identified themes, there was a prevalent feeling of isolation among the adolescents, as well as a strong need for transparency, preparation, and honest information.

The adolescents in this study were exposed to a confrontation with death that challenged their sense of security. Yalom [36] describes the existential givens that represent the underlying conditions of human existence that everyone, regardless of context, faces. These include death, freedom, isolation, and meaninglessness [36]. Critical illness had numerous effects on the adolescents in our study, such as a lack of opportunity to prepare themselves, experiencing shock and confusion, a sense of loss of control, disruption of routines, and an increased emotional burden. Friedman [37] asserts that the most important institution in a person’s life is the family. All the adolescents in our study experienced strong bonds with their relatives, with most having a parent as the ICU patient. With the family viewed as a unit in which all members feel a sense of belonging, critical illness acts as a separation that destabilises the foundation and renders life uncertain [38].Baumeister and Leary view ‘belonging’ as a fundamental human need for interpersonal connections. They argue that people have an innate drive to establish meaningful relationships, which impact emotions, thoughts, and behaviours. Satisfying this need promotes mental and physical well-being, while a lack of belonging can lead to distress and negative health effects [38].

Friedman [37] describes how developmental needs of healthy family members go unmet and family patterns become dysfunctional during times of stress such as acute illness or death in the family. The adolescents in our study maintained strong bonds with their relatives. These bonds were disrupted when a relative was admitted to the ICU, and the adolescents experienced this situation as very stressful. Severe illnesses like cancer, chronic diseases, mental illness, addiction disorders or substance use have a significant impact on the adolescents relational and personal strain and growth [39], where most studies conducted reveals negative influence on adolescents life [40–42]. The adolescent relatives in the ICU differs from other groups of adolescents because the critical illness of their relative typically occurs unexpectedly, often accompanied by uncertain prognoses. These adolescents experience significant psychological distress and are at high risk of developing anxiety, depression, and PTSD [20, 22]. In order to prevent the consequences that critical illness has on the family, including adolescents, we believe that the FCC framework can contribute to a sense of belonging through respect, participation, information-sharing and collaboration [18]. This aligns with the findings of our study, which indicate that adolescents do not feel welcome in the ICU; they desire and expect to be present, need more information, and wish to be included. Family-centred care is increasingly recognised as a quality criterion in modern ICUs [16–18].

There is strong evidence that children and adolescents of seriously ill parents need information and support [1, 24, 40, 43]. Nevertheless, the perspective of children and adolescents is lacking when a relative is seriously ill. Moreover, studies have concluded that children and adolescents continue to experience being overlooked as important contributors and are left without any clear understanding of the relative’s situation [1, 24]. The adolescents in our study often felt invisible, but had a strong need to be seen and acknowledged, which aligns with experiences reported by MacEachnie, Larsen [1]. They reported that both parents and HCPs tend to shield them from the realities of their situation. In our study, the adolescents simultaneously shielded their parents from how they truly felt, believing their parents had enough to deal with themselves and lacked the capacity to care for them. Appearing ‘fine’ seemed safer for our adolescents than expressing any needs. Studies have demonstrated that adolescent relatives in palliative care face uncertainty and fear. In addition, they often withhold their emotions and questions about the illness to avoid overburdening their parents or because they fear their feelings will not be acknowledged [44, 45]. The adolescents in our study experienced being in the middle of it all, while at the same time being outside. This experience led to feelings of isolation and invisibility. CCNs have a core responsibility to see and acknowledge all relatives. However, the attitudes of nurses regarding the importance of family in nursing care vary significantly in European countries [46]. A few of the adolescents in our study experienced being cared for and informed by the CCNs and HCPs in the ICU, but most felt unseen. The barriers that exist when it comes to the involvement of relatives lead individual nurses to make unilateral decisions about whether to include or exclude relatives from the care process [47].

It is crucial that HCPs are willing to truly acknowledge adolescents and engage in a non-judgemental dialogue with them [1, 3]. The adolescents in our study stated that simple gestures such as a smile or someone asking, ‘How are you?’ would suffice. Measures such as this could have a positive impact on contributing to an environment of belonging for adolescent relatives in the ICU.

Fifteen years ago, the Norwegian legislation was amended to protect and safeguard children and adolescents as relatives [25]. However, our study demonstrates that we are still failing to provide these children and adolescents with the individual support and information that they both require and are entitled to receive. The same findings apply in the case of Sweden, where the rights of children as relatives are just as strong as they are in Norway [23].

While FCC is implemented in many ICUs throughout the world [48], there is a need for a greater focus on recognising relatives as an integral part of resource allocation and patient care planning. Mindful of adolescents’ need to belong, the care given to adolescent relatives can be improved. In Nordic ICUs, children and adolescents do not receive routine follow up [49]. Visits to the ICU by underage children and adolescents remain a controversial issue [24]. However, research has indicated that regardless of visitation status, most children and adolescents exhibit symptoms of anxiety and depression. Visits have been demonstrated to be potentially traumatic [21], with adolescents experiencing shock, especially if they are unprepared for seeing the ICU patient. Yet the absence of such visits is perceived as more detrimental for adolescents [3]. In accordance with FCC, families should be afforded open and flexible presence and participation [16]. The adolescents in our study desired flexible visiting hours and encouraged ICU staff to support and invite them to visit, which aligns with the findings of Fergé [20]. Flexible visiting has been associated with a positive impact on family members in ICUs [50].

Although visitor restrictions and visitation policies are changing [23] there are still barriers that prevent children and adolescents from visiting the ICU [24]. Nurses often rely on their personal judgement rather than following guidelines or evidence regarding visits by children and adolescents [12]. Parents are fearful and they want to protect their children and adolescents by keeping them out of the ICU [24, 43]. The adolescents in our study expressed a perceived necessity for presence and proximity to the patient. They regarded it as self-evident that they should visit their relatives in the ICU. Despite this, children still rarely visit ICUs [49]. Furthermore, the adolescents in our study expressed a clear and explicit desire for more support in the form of being prepared by ICU staff before their first encounter with the ICU and the initial visit with their relative. This is in line with the findings of Lamiani [3], who stress the importance of preparation and facilitation of the visit. Communication between HCPs in the ICU and family members is crucial [8, 16]. Most of the adolescents in our study experienced being excluded from communication, prompting them to request updates and continuous information. Frivold and Ågård [49] identified potential for improvement in the inclusion of children and adolescents through active communication and individual information. Moreover, Knutsson and Golsäter [23] found that the information provided by nurses to children and adolescents in the ICU was superficial and needed to be more individualised and comprehensive.

In our study, those adolescents who were prepared and received information through conversations in the ICU experienced much less concern than others. Supporting adolescent relatives in the ICU requires providing tailored information, as studies demonstrate that adolescents often struggle to comprehend the information they receive [23]; or they have received some information, but desire more [40]. This sense of trust and understanding can empower adolescents to express their concerns and ask questions, leading to a more supportive and effective healthcare experience for both the adolescents and their families. Nurses face significant challenges when caring for adolescents in various healthcare contexts [51]. CCNs should have a central role in supporting adolescents. None of the adolescents in our study had a clear understanding of the role of the CCN role, even though there is a 1:1 or 2:1 nurse-to-patient ratio in Norwegian ICUs [52]. This means that all visiting adolescents must have met the CCN. CCNs and doctors demonstrate an excellent knowledge of family needs, but only a few can prioritise them, thus highlighting a gap between knowledge and its practical application [48].

### Limitations

The study has limitations that warrant consideration. Most of the adolescents in the study were girls, with an average age of approximately 17 years. A more heterogeneous sample might yield more nuanced findings. In addition, the inclusion of more boys could offer different perspectives or gendered aspects. Moreover, all adolescent participants were of white Norwegian/North European ethnicity. The absence of participants from other ethnic, religious, or cultural backgrounds limits the study’s diversity. Furthermore, the severity of the relatives’ critical illness varied; some stayed in the ICU for only three days, some became long-term ICU patients, while others died in the ICU. Given that this study was conducted within the context of Norwegian healthcare, its transferability to other healthcare settings may be limited. In addition the descriptive nature of the study might represent a limitation related to transferability. The sample in the present study primarily represents the parent/child relationship. The perspectives of siblings and grandchildren are not specifically elaborated.

## Conclusions

The importance of understanding adolescents’ experiences as relatives of ICU patients lies in addressing their unique challenges and emotional burdens, ensuring they are visible, prepared, informed, and supported in a potentially overwhelming environment. They experience significant uncertainty and distress, largely influenced by the separation from their relatives. The intimidating and technical nature of the ICU environment often provokes shock reactions in adolescents, who frequently feel overlooked and in need of a more welcoming and supportive atmosphere. Utilising these perspectives from Norwegian adolescents makes them more applicable, providing healthcare professionals with tools to assist adolescent relatives in the ICU. This is vital research for the future of adolescents and can contribute to changing practice. CCNs and other healthcare professionals in Norwegian ICUs have the potential to improve in terms of complying with legislation regarding children and adolescents as relatives. To achieve the objectives of FCC and involve adolescents as relatives in ICUs, CCNs and other healthcare professionals must remain aware of their responsibilities. They should do this in compliance with legislation and with a view to fostering a sense of belonging that minimises the traumatic ICU experience. Moreover, health authorities should focus more strongly on prioritising the resources needed to fulfil this responsibility.

## Supplementary Information

Below is the link to the electronic supplementary material.

Supplementary Material 1

## Data Availability

Data availabilityThe data were collected in the participants’ native language, and consent has been obtained for anonymized data to be published, but no consent has been made for data to be shared.Consent for publicationNot applicable.
